# Computer Vision Based Automatic Extraction and Thickness Measurement of Deep Cervical Flexor from Ultrasonic Images

**DOI:** 10.1155/2016/5892051

**Published:** 2016-02-01

**Authors:** Kwang Baek Kim, Doo Heon Song, Hyun Jun Park

**Affiliations:** ^1^Department of Computer Engineering, Silla University, Busan 617-736, Republic of Korea; ^2^Department of Computer Games, Yong-in Songdam College, Yongin 449-040, Republic of Korea; ^3^Department of Computer Engineering, Pusan National University, Busan 609-735, Republic of Korea

## Abstract

Deep Cervical Flexor (DCF) muscles are important in monitoring and controlling neck pain. While ultrasonographic analysis is useful in this area, it has intrinsic subjectivity problem. In this paper, we propose automatic DCF extractor/analyzer software based on computer vision. One of the major difficulties in developing such an automatic analyzer is to detect important organs and their boundaries under very low brightness contrast environment. Our fuzzy sigma binarization process is one of the answers for that problem. Another difficulty is to compensate information loss that happened during such image processing procedures. Many morphologically motivated image processing algorithms are applied for that purpose. The proposed method is verified as successful in extracting DCFs and measuring thicknesses in experiment using two hundred 800 × 600 DICOM ultrasonography images with 98.5% extraction rate. Also, the thickness of DCFs automatically measured by this software has small difference (less than 0.3 cm) for 89.8% of extracted DCFs.

## 1. Introduction

Neck pain is very common complaint affecting up to 70% of individuals at some point of their lives [1]. Once an individual develops neck pain, it is reported that there is a 1 in 3 chance that he or she will develop chronic symptoms lasting longer than 6 months, and the incidence of mechanical neck disorders appears to be increasing [2]. Clinical neck pain is associated with impairment of muscle performance and the functional impairments associated with neck pain and the cause-effect relationships between neck pain and motor control are well investigated [3].

The Deep Cervical Flexor (DCF) muscles including longus colli muscle, longus capitis, rectus capitis inferior, and rectus capitis lateralis have major roles in maintaining cervical lordosis and providing cervical joint stabilization [3, 4]. It has been theorized that when muscle performance is impaired, the balance between the stabilizers on the posterior aspect of the neck and the DCFs will be disrupted, resulting in loss of proper alignment and posture, which is then likely to contribute to cervical impairment [5].

A strong linear relation between the electromyographic amplitude of the DCF muscles and the incremental stages of the craniocervical flexion test for control and individuals with neck pain was reported [6]. Another study showed that patients with neck pain disorders have an altered neuromotor control strategy during craniocervical flexion characterized by reduced activity in the DCFs and increased activity in the superficial flexors usually accompanied by altered movement strategies. Furthermore, they display reduced isometric endurance of the DCFs [7].

These observations prompted the use of the craniocervical flexion action for retraining the DCF muscles for neck pain patients. Specific training of the DCF muscles in women with chronic neck pain reduces pain and improves the activation of these muscles, especially in those with the least activation of their Deep Cervical Flexors before training [8]. The recruitment pattern of the DCF and sternocleidomastoid is investigated during the craniocervical flexion test, using ultrasonography [9, 10], and it is further developed into an exercise program with tools [11].

Using ultrasound image in muscle analysis is appropriate for its noninvasive, inexpensive, real time responding capabilities [12]. However, its limitations are often pointed out; sonographic images are dependent on the qualities of equipment and skills of expertise; thus, the diagnosis often misleads to subjective judgments [13]. Thus, we need an automatic image segmentation and identification tool for anatomical landmarks that can eliminate such subjectivity in the image analysis [14].

Unfortunately, there is almost no directly related research for such an automatic DCF extractor/analyzer by computer vision yet. A recent study tried to give an automatic segmentation of cervical vertebrae from X-rays [15] but not related to muscles of our interests. Probably, our previous research that automatically detects sternocleidomastoid and longus capitis/colli is the only one to consider [16].

In this paper, we extend our previous automatic cervical muscle extractor/analyzer to accommodate with DCFs which are longus capitis (Lcap) and longus colli (Lcol). While certain part of extracting techniques is common to the previous study that extracts sternocleidomastoid and longus capitis/colli, the overall treatment of target muscle DCF is different from that of other related muscles due to the muscle characteristics and the location of the muscles.

The main methodological difference between our previous study [16] and this work is whether the system extracts the cervical vertebrae directly or not. The brightness contrast between DCF and related fascia is much weaker than between SCM and its related fascia in ultrasonography. Also, the cervical vertebrae, located directly below the DCF, have similar brightness values to those of DCFs. Thus, when we infer the location of cervical vertebrae using the slope of lower boundary lines of DCFs in [16], the key point to measure the thickness of DCF is not correctly extracted especially when the contrast between the fascia/bones and DCFs is low. Thus, in this paper, we extract cervical vertebrae directly and the key points are induced based on the location of cervical vertebrae.

## 2. Overall Procedure

**Figure 1:**
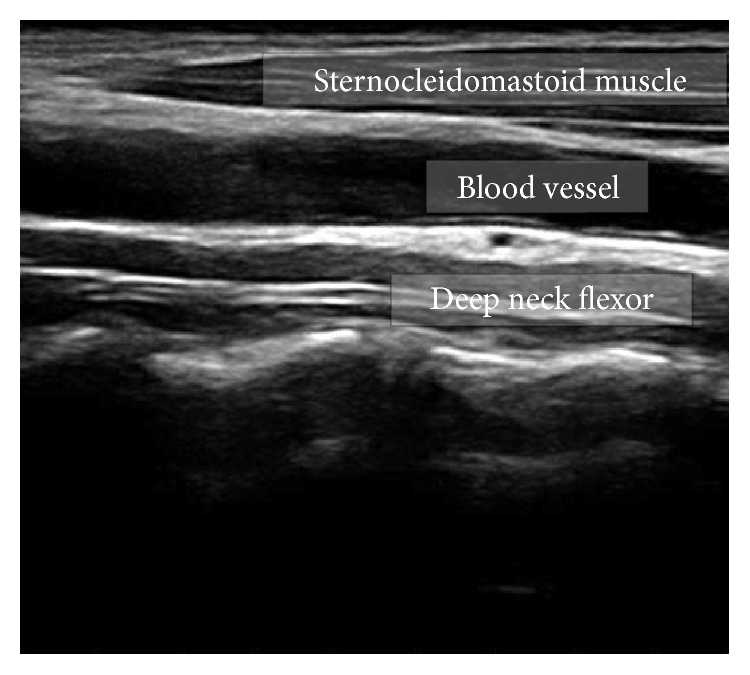
ROI of ultrasound image.

All the digital images used in this study are acquired and stored in DICOM (digital imaging and communications in medicine) standard format. DICOM is a form to declare image transfer, structure, and related information. In region of interest (ROI) part of the image shown in Figure 1, there is a blood vessel and two muscles are located above and below the vessel. The muscle above blood vessel is the sternocleidomastoid and the muscle below the blood vessel is the DCFs. DCFs have irregular form of border with cervical spine at the bottom and is unclear being far from the center. The brightness of the muscle is usually lower than that of the fascia and the spine. The fat area that has relatively high brightness in the muscle area should be extracted with the muscle.

**Figure 2:**
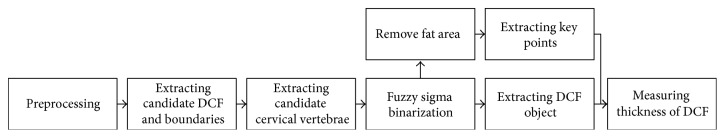
Overall system diagram.

Figure 2 summarizes the overall vision based system to extract and measure the thickness of DCFs automatically.

## 3. Extracting Deep Cervical Flexor

**Figure 3:**
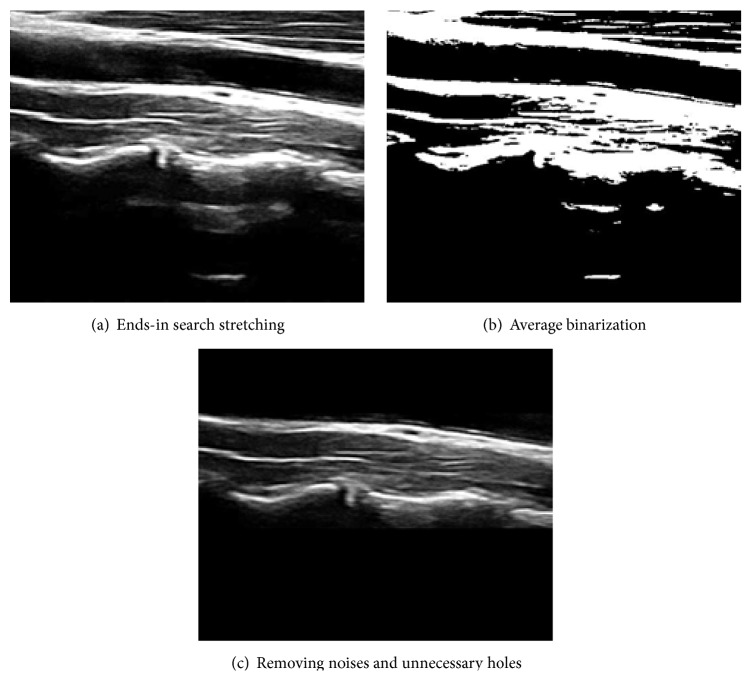
Extracting Deep Cervical Flexor candidate.

From this ROI image that contains only muscles, fasciae and spines, we try to extract candidate DCF by applying a series of image processing algorithms as shown in Figure 3.

The ends-in search stretching method is a normalization process that enhances the intensity contrast to differentiate two areas more clearly as shown in Figure 3(a). Formula (1) explains ends-in search stretching

(1) Px,y=0Gx,y≤Min255×Gx,y−MinMax−MinMin<Gx,y<Max255Gx,y≥Max,

where Min and Max denote the maximum and minimum intensity value of the given image and *G*(*x*, *y*) is the intensity value of the pixel at (*x*, *y*) coordinates in the original image and *P*(*x*, *y*) is the resultant intensity value after ends-in search stretching.

The rationale of using ends-in search stretching is as follows.

Due to the scattering effect of ultrasound technology, there might be blurring that gives difficulty in discriminating fascia, muscles, and cervical vertebrae. Thus, we need a contrast enhancing mechanism with minimal information loss.

**Figure 4:**
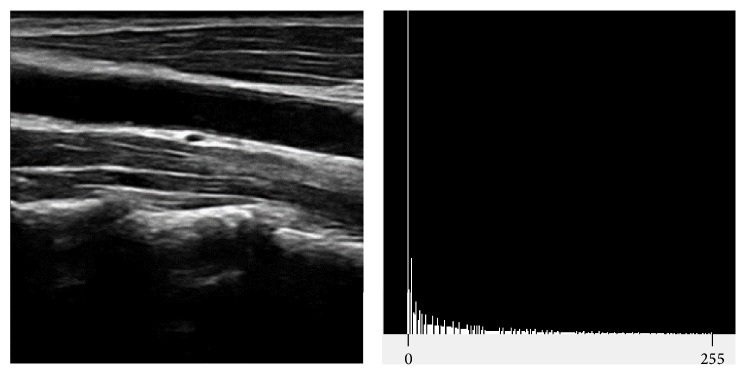
Cervical vertebrae sonography and histogram.

In our method of extracting DCF, we first extract the fascia lines and cervical vertebrae that are relatively brighter than others and SCM and DCF are extracted by using location characteristics related to the blood vessel. A typical craniocervical sonography has largely skewed distribution of intensity toward zero as shown in Figure 4 and those low intensity pixels have slim chance to be related to objects of our concern. Ends-in search stretching, a type of normalization process, is then advantageous than other methods such as filtering since it preserves the relative characteristics of the intensity distribution. However, it needs proper noise removal process afterwards.

Next, we apply average binarization to the enhanced image shown in Figure 3(b). Then, we apply Blob algorithm [17] to group pixels into objects. Figure 3(b) contains unnecessary noise objects. As explained in Section 5 in detail, we obtained 200 images from 100 healthy subjects during a neck muscle endurance test. We know that DCFs in consideration have specific morphological characteristics that we can use in noise removal process. In order to quantify such characteristics that can be generalized, we choose 50 random samples from 200 image population and observe any size or location related features that are common to apparent noise objects in this situation.

As shown in Figure 1, there exists blood vessel in between SCM and DCF; thus, two muscles are apart from a certain distance. From our samples, the distance between two muscle objects in *y*-axis is no less than 8.5% of the ROI height. Also, we know that the lower fascia boundary lines and bones have a long curved shape; thus, it is not at the skewed location to the left in any of our obtained images. Thus, we can set up a safe *x*-axis constraint such that an object that is no longer than the half of the ROI width starts with the very leftmost position (10% of the ROI width). Formula (2) below shows our noise removal criteria used in this paper

(2) $$\begin{array}{c} \begin{array}{c} \text{if }\left(\left(S\left(O\right)<0.085\times S\left(ROI\right)\right)\right.\text{OR}\\ \;\;\left(O.left<0.1\times \mathrm{Width}\left(ROI\right)\right.\text{AND}\\ \;\;\left.\left.\mathrm{Width}\left(O\right)<0.5\times \mathrm{Width}\left(ROI\right)\right)\right)\\ \text{then }\mathrm{Remove}\left(O\right), \end{array} \end{array}$$

where *S*(*O*), *S*(ROI) denote the size of the object in consideration and that of ROI area, respectively, and* O.*left denote the *x*-coordinate of the leftmost boundary of the object *O* and Width(*X*) is the function of returning the width of object *X*.

**Figure 5:**
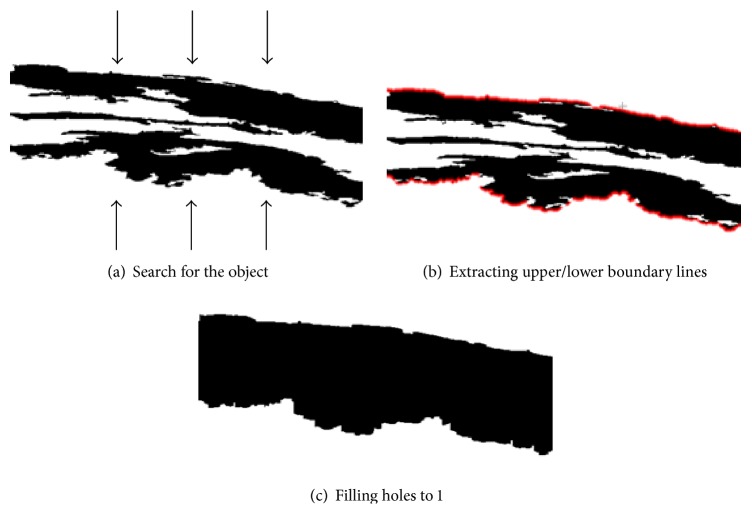
Filling holes by searching boundaries of DCF.

There might be some unnecessary holes in between the upper and lower boundary lines of candidate DCF. In order to fill such holes systematically, we search upper and lower boundary object by searching from the top and the bottom of the ROI area as shown in Figure 5. That results in an image like Figure 3(c).

Since we apply many image processing algorithms that enhance the contrast of the brightness for purposes, there might exist cases where the boundary lines are disconnected in part. The first simple treatment for restoration is to fill brightness values of pixels with 255 if they are neighbors of 255-valued ones and makes the line connected. The lower boundary lines have relatively complex shape; thus, we apply 4-directional contour search [17] to determine the boundary lines. However, again, it may have discontinued part.

Thus, we apply cubic spline interpolation to reconnect lower boundaries. The necessary conditions of cubic spline interpolations are as follows:

(3) Sn−1′′Pn=S0′′P0=0,Si−1′′Pi=Si′′Pii=1,2,…,n−1,Si−1′Pi=Si′Pii=1,2,…,n−1,Si−1Pi=SiPi=yii=1,2,…,n−1,

where *S*_*i*_ denotes a Spline function and *i* is the coordination on the boundary and *n* denotes the number of coordination on the boundary and *y*_*i*_ is the functional result of *P*_*i*_. Then, the Spline function *S* is defined as follows:

(4) $$\begin{array}{c} S_{i}\left(\;P\;\right)=a_{i}+b_{i}\left(\;P-P_{i}\;\right)+c_{i}{\left(\;P-P_{i}\;\right)}^{2}+d_{i}{\left(\;P-P_{i}\;\right)}^{3}, \end{array}$$

where *a*, *b*, *c*, and *d* are constants satisfying Spline function conditions [18].

**Figure 6:**
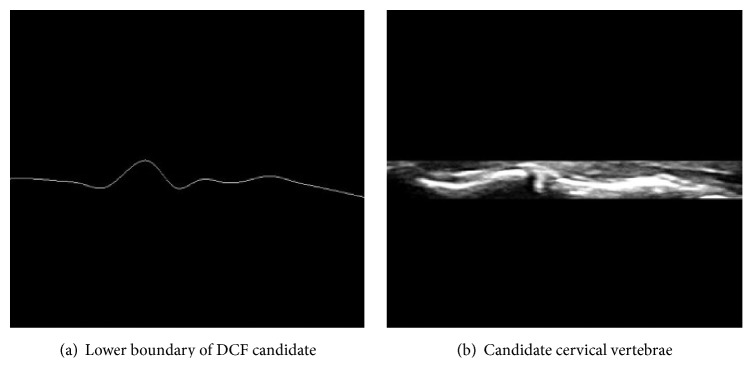
Candidate cervical vertebrae extraction with image restoration.

Figure 6 demonstrates the extracted reconnected lower boundaries of DCF Figure 6(a) and the overall shape of the extracted candidate of cervical vertebrae Figure 6(b).

The next step is the binarization procedure. The main purpose of binarization is to find an optimal thresholding value to discriminate the target organ from background of the input image. Our goal is to find the upper bound and lower bound lines of DCF area. Unfortunately, the brightness contrast near DCF area is not clear in general. Thus, we need more detailed computationally heavy binarization procedure called fuzzy sigma binarization [19].

**Figure 7:**
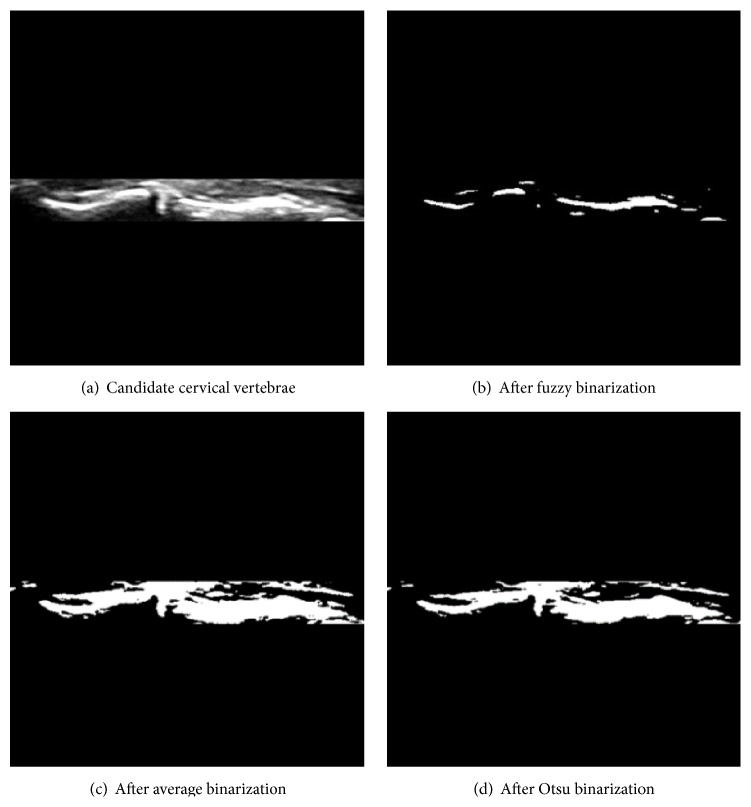
Different binarizations in candidate cervical vertebrae extraction.

The major reason that we adopt computationally heavy fuzzy binarization over simple average binarization or Otsu binarization [20] is the environmental characteristic of the near DCF area. Since cervical vertebrae, DCFs, and related fascia have similar brightness values, the average binarization may include extracting false positive objects in such a low contrasted environment. Otsu binarization that assumes the image contains two classes of pixels following bimodal histogram and searches for the optimum threshold separating the two classes so that the interclass variance is minimal is another alternative, but, in this environment, the cervical vertebrae are relatively brighter than nearby muscles and fascia; thus, the interclass variance should not be minimal since we want to extract cervical vertebrae in this case. Thus, fuzzy sigma binarization that is designed to adapt the sensitivity of environment (brightness contrast) and qualitative membership decision by fuzzy membership function is our choice.  Figure 7 demonstrates the effects of using different binarization methods in this environment.

The fuzzy membership function of our fuzzy sigma binarization is defined as follows.

Let *P*_Max_, *P*_Min_, and *P*_Mid_ be the highest and lowest brightness value of the pixel and the average of *P*_Max_ and *P*_Min_, respectively. Then, the membership function is defined as follows.

**Step 1 .**

Consider the following:

(5) PMinF=PMid−PMinPMaxF=PMax−PMid.

**Step 2 .**

Consider the following:

(6) $$\begin{array}{c} \begin{array}{c} if\;\left(P_{Mid}>P_{Min}+0.75\left(P_{Max}-P_{Min}\right)\right)\;\;then\;\;P_{Mid}^{F}\\ \;\;=255-P_{Mid}\\ else\;\;P_{Mid}^{F}=P_{Mid}. \end{array} \end{array}$$

**Step 3 .**

Consider the following:

(7) $$\begin{array}{c} \begin{array}{c} if\;\;\left(P_{Mid}^{F}>P_{Max}^{F}\right)\;\;then\\ \;\;if\;\;\left(P_{Min}^{F}>P_{Mid}^{F}\right)\;\;then\;\;\beta =P_{Mid}^{F}\\ \;\;else\;\;\beta =P_{Min}^{F}\\ else\;\;if\;\;\left(P_{Max}^{F}>P_{Mid}^{F}\right)\;\;then\;\;\beta =P_{Mid}^{F}\\ else\;\;\beta =P_{Max}^{F}. \end{array} \end{array}$$

**Step 4 .**

Calculate the normalized *P*_Min_^new^ and *P*_Max_^new^

(8) PMinNew=PMid−β,PMaxNew=PMid+β.

Step 2 is designed to compensate overly brightly filmed image due to filming environment while classifying *P*_Mid_^*F*^ based on the three quarters of brightness contrast.

The membership degree of a pixel by sigma fuzzy binarization is as follows within the interval [*P*_Min_^New^, *P*_Max_^New^]:

(9)  if  P≤PMin⁡New,  then  uP=0, if  PMinNew<P<PMidNew,  then  uP=P−PMinNewPMidNew−PMinNew, if  PMidNew≥P,  then  uP=1.

**Figure 8:**
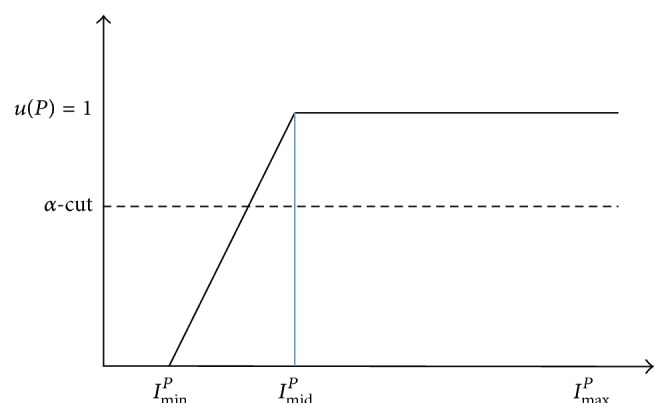
Membership function for sigma fuzzy binarization.

The membership degree *u*(*P*) is applied to the* a-cut* (set to 0.5 in this paper since there is no prior information or preferences.) for the binarization such that the pixel value would be set to 255 if *u*(*P*) ≥ 0.5 and 0 otherwise.  Figure 8 shows the membership function of sigma type.

**Figure 9:**
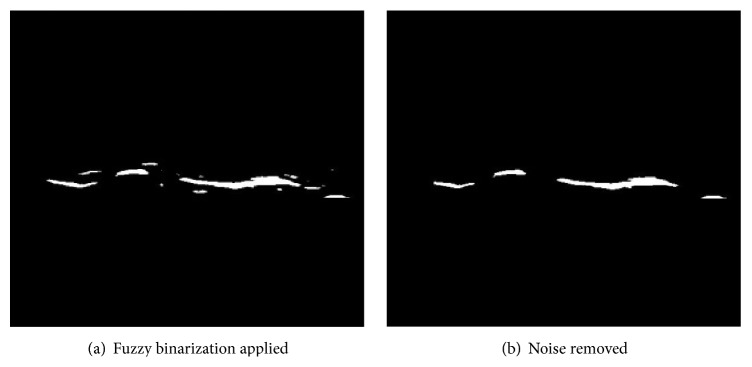
DCF area treatment for connectivity.

Again, we apply Blob algorithm to remove unnecessary noises and apply expansion operation to restore small discontinuity if exists. The result is shown in Figure 9.

**Figure 10:**
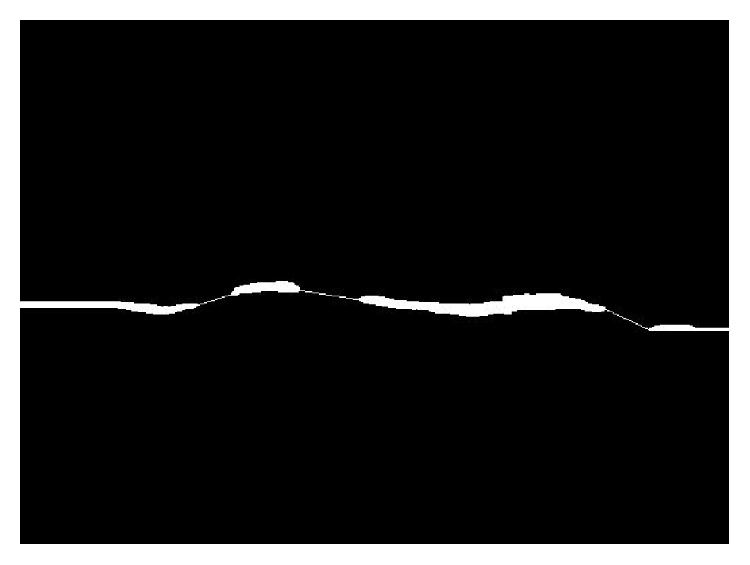
Boundary reconnection of cervical vertebrae by DDA algorithm.

After expansion operation, we can extract the lower boundaries of DCF using relative location information among objects appearing in the cervical vertebrae area. Subcutaneous fat objects existing above DCF will be removed for the simplification of further analysis. Possible disconnections caused by fat removal are restored by simple Digital Differential Analyzer (DDA) algorithm [19] that is a type of simple linear interpolation of slope *m* between given intervals using the following equation:

(10) Qx,y=mx−x1+y1,m=y2−y1x2−x1,

where *Q*(*x*, *y*) denotes the coordinates of a pixel in between the left side of the extracted object and the right cervical vertebrae. The effect of DDA algorithm can be shown in Figure 10.

The upper bound lines of Figure 10 are the lower boundary lines of DCF area. Since all other objects such as subcutaneous fat are removed already, it is simple to find upper bound lines of Figure 10. However, there might be small disconnections. Again, cubic spline interpolation defined as (3) and (4) is applied to compensate lost information.

**Figure 11:**
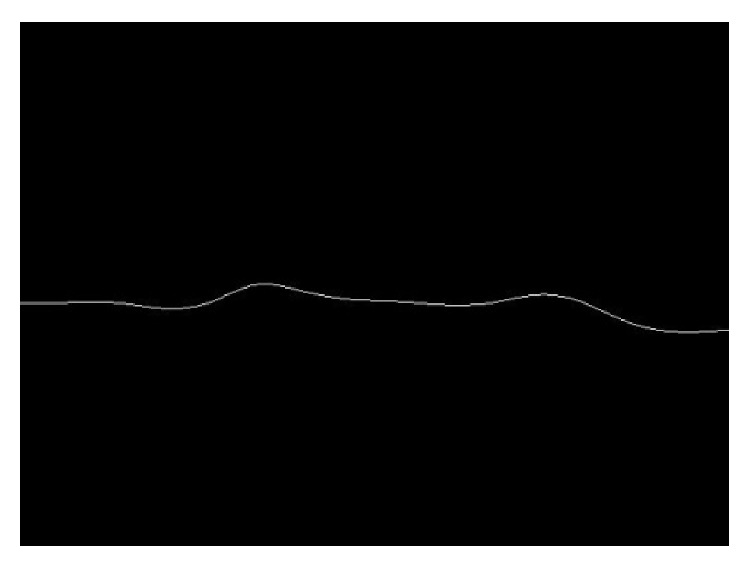
Extracting lower boundaries of DCF.

**Figure 12:**
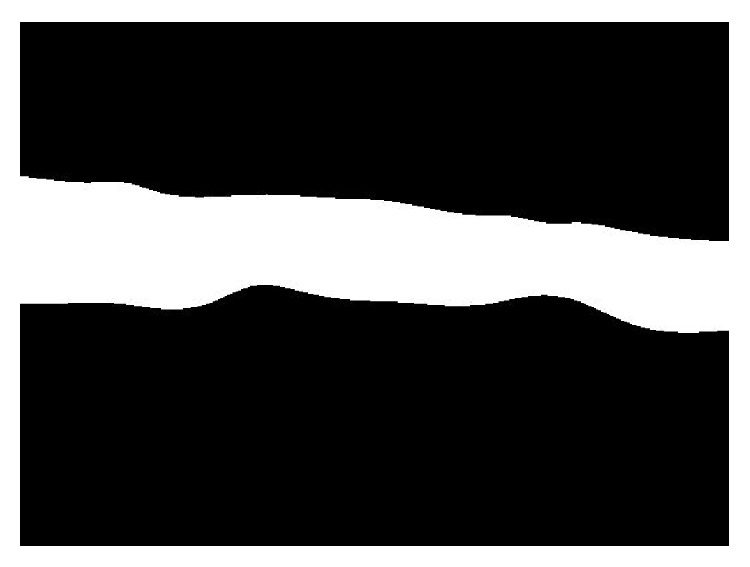
Final extraction of DCF area.

Figure 11 demonstrates the final extraction of lower boundaries of DCF. The final extraction result of DCF is shown in Figure 12.

## 4. Measuring Thickness of Deep Cervical Flexor

Among many possible characteristic features of DCF, the thickness is the most fundamental one, but it is also the main source of subjectivity in measuring morphometric features. Computer vision based approach like us aims to locate measuring key points accurately to achieve the automaticity of capturing such measuring standards to avoid subjectivity as much as possible. In our proposed method, there are three cervical vertebrae in the image and the measuring key point is set to be the rightmost point of the leftmost cervical vertebrae object. Then, from that key point, two other measuring points are chosen to be 1 cm left and right to the key point. The thickness is then computed as the average of those three vertically measured lengths passing through the DCF. The details of such measuring process are as follows.

The leftmost cervical vertebra should be located at the left part of the image. However, when we apply the labeling procedure to obtain target objects, if the leftmost cervical vertebra is found at the right part of the given image, that means the real leftmost cervical vertebra is lost as noise during preprocessing. Then, we apply weighted image restoration process to the image after fuzzy sigma binarization (Figure 9(a)) as shown in formula (11) to avoid unwanted object removal

(11) α=1.0+x−x12+y−y121000,Px,y=Px,y×α,

where *α* is the weight and this formula gives more weight to the points of the far left points from the center point (*x*_1_, *y*_1_). This compensation process is designed to avoid candidate cervical vertebra object located at the left side of the image.

**Figure 13:**
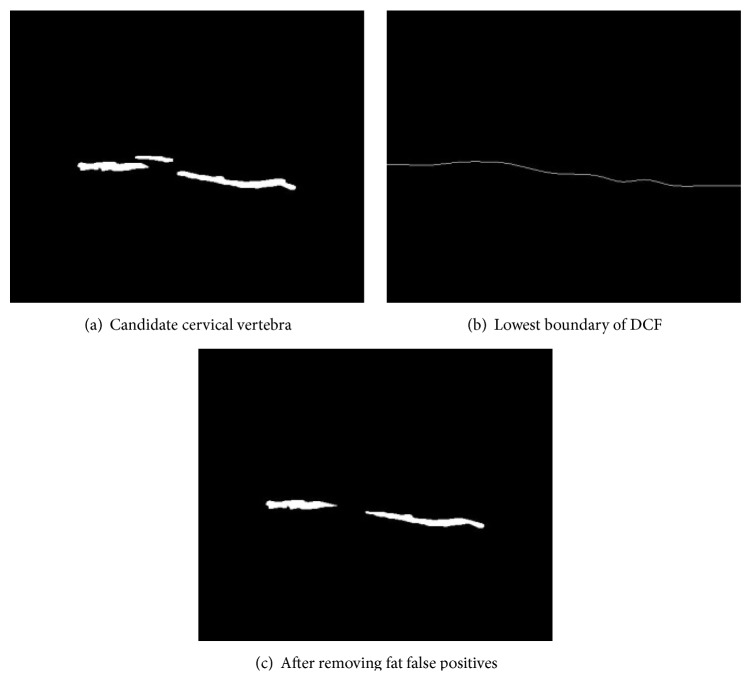
Fat area noise removal.

After applying Blob algorithm and object labeling procedure, if a candidate cervical vertebra is located above the lowest line of DCF, it is probably the fat area falsely regarded as cervical vertebra; thus, we remove such false positives as shown in Figure 13.

**Figure 14:**
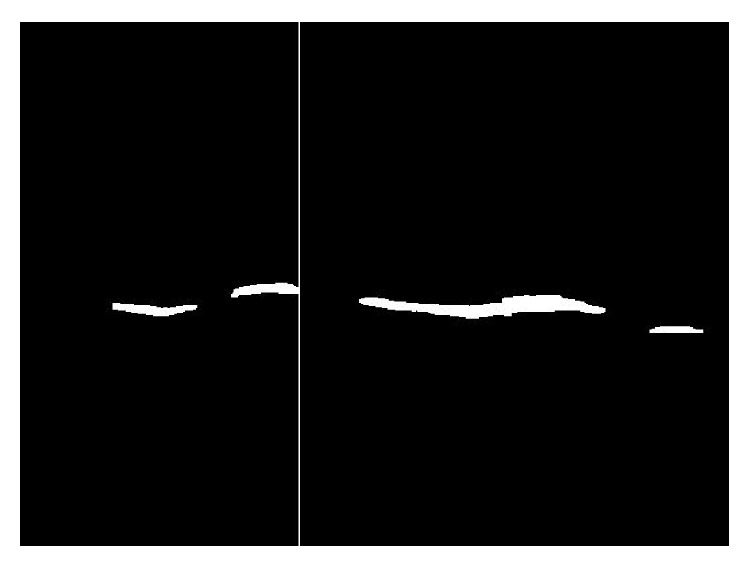
Key point extraction.

After such compensation process, the measuring key point is set to be the rightmost point of the leftmost cervical vertebrae object as shown in Figure 14.

## 5. Experiment

The proposed method is implemented with C++ under Microsoft Visual Studio 2010 on the IBM-compatible PC with Intel Core i7-2600 CPU @ 3.40 GHz and 4 GB RAM. The experiment uses two hundred (200) 800 × 600 size DICOM format ultrasound images. One hundred healthy subjects of age in their 20s and 30s participated in this experiment.

We measured neck muscle size during a neck muscle endurance test. The thickness of neck muscle was assessed using ultrasonography (MyLab 25GOLD) with 12 MHz linear probe during neck muscle endurance tests. Sternocleidomastoideus (SCM) and the deep cervical muscles, which are longus capitis (Lcap) and longus colli (Lcol), were measured laterally (right and left side) at the level of C4. Subjects performed a craniocervical flexion test (CCFT). The CCFT is commonly used in the physiotherapeutic assessment of neck muscle strength and endurance. During the CCFT, subjects were instructed to perform a nodding movement, representing the craniocervical flexion, in five incremental levels, from 22 to 30 mmHg: 22, 24, 26, 28, and 30 mmHg. The subjects were asked to maintain the test positions for as long as possible. Each subject was asked to sit upright in an examination chair with their arms resting on their thighs. A head was fixed to maintain the head and neck in the neutral position. The C4 segmental level was chosen to optimize the image field for CCFT and thus infer the coordination between the three muscles in the midcervical spine. At the C4 level, we captured different parts of the Lcol, Lcap, and SCM.

**Figure 15:**
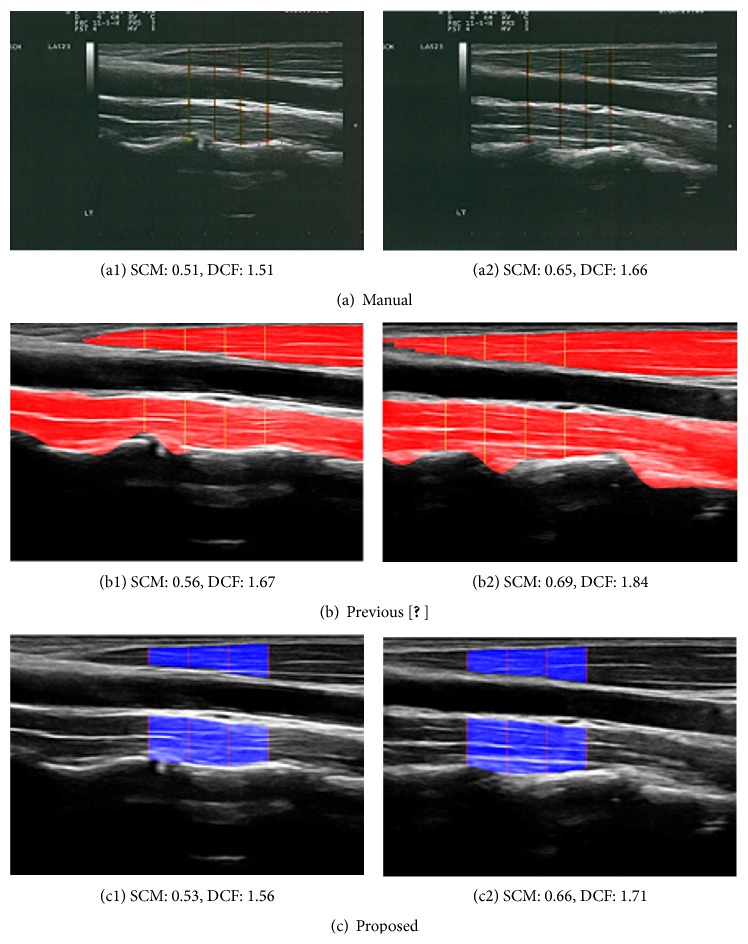
Comparison of muscle thickness measurements (cm).

Figure 15 visually demonstrates the result of the proposed automatic DCF extractions (c1 and c2) compared with physical therapist's manual inspections (a1 and a2) and our previous attempts (b1 and b2) for the same images. One can verify that our vision based automatic software extracts almost identical key points/area of target muscles and the magnitude of difference between automatic measurement and manual measurement becomes much smaller than that of previous attempt. In our experiment, two medical experts are involved and the thickness of the manual inspection is taken as the average of the two human experts' evaluations.

As summarized in Table 1, the proposed method is much better than the previous attempt [16] in DCF extraction in sensitivity (SS), where TP, TN, FP, and FN denote the true positive, true negative, false positive, and false negative, respectively.

Due to the image acquisition process explained above, there is no image that does not contain DCF and SCM. Thus, there is no TN or FN. The decision to classify the result of automatic extractions is based on the agreement of two field experts involved in this experiment.

Table 2 also shows the improved sensitivity in SCM extractions.

Also, after measuring the thickness, we compare our result with the previous attempt as the absolute difference from human experts' measurement (error). The results for the error magnitude of thickness measurement for DCF and SCM are summarized in Tables 3 and 4, respectively.

While much improved compared to the previous attempt [16], the distribution of error magnitude in DCF thickness measurement shows that there is a room for the improvement in the future. At least, the proposed method is successful in extracting DCF and SCM accurately and the sensitivity of such extractions is much more improved compared to the previous attempt.

## 6. Conclusion

In this paper, we propose a vision based fully automatic method to extract DCF muscles (longus capitis/colli) and SCM with measuring the thickness from cervical vertebrae ultrasound images. DCF muscles are important in controlling/monitoring the neck pain and/or develop efficient/effective rehabilitative training procedures. However, existing clinical method using ultrasonography often causes operator effect, a subjective judgments of muscle extraction and associated morphometric features measurement and analysis. Our attempt shown in this paper is to avoid such subjectivity as much as possible and aims to assist human medical experts in DCF and SCM analysis.

Algorithmically, our previous attempt [16] that extracts and analyzes sternocleidomastoid and longus capitis/colli automatically often suffers from the distraction of ultrasonography. The major contribution of this paper is to overcome that problem with careful image processing considering morphological characteristics of DCFs (shape and location information) and extracting cervical vertebrae explicitly in the process. Many image processing algorithms are involved such as Blob for noise removal and ends-in search stretching for image enhancement and 4-directional contour search for boundary detection and cubic spline interpolation for complementing disconnected lines and the fuzzy sigma binarization to control the low brightness contrast environment.

The experimental result using 200 real-world cervical vertebrae DICOM ultrasound image verifies that almost all (98.5%) input images are well extracted and the thickness is automatically measured with low error magnitude with respect to those of human experts' decision (≤0.3 cm) in most cases (89.8%).

## Figures and Tables

**Table 1:** DCF extraction performance evaluation.

Success rate	DCF (longus capitis/colli)				
	TP	TN	FP	FN	SS
Previous [16]	147	0	53	0	73.5%
Proposed	197	0	3	0	98.5%

**Table 2:** SCM extraction performance evaluation.

Success rate	SCM				
	TP	TN	FP	FN	SS
Previous [16]	169	0	31	0	84.5%
Proposed	198	0	2	0	99.0%

**Table 3:** Error magnitude of DCF (longus capitis/colli) thickness (# of successes/# of tried images).

	<0.1 cm	<0.2 cm	<0.3 cm	≥0.3 cm
Previous [16]	3/147 (2.0%)	32/147 (21.8%)	72/147 (49.0%)	40/147 (27.2%)

Proposed	13/197 (6.6%)	59/197 (29.9%)	105/197 (53.3%)	20/197 (10.2%)

**Table 4:** Error magnitude of SCM thickness (# of successes/# of tried images).

	Error < 0.1 cm	Error ≥ 0.1 cm
Previous [16]	141/169 (83.4%)	28/169 (16.6%)

Proposed	175/198 (88.4%)	23/198 (11.6%)
